# 

**Published:** 1860-11

**Authors:** 


### ORIGINAL COMMUNICATIONS.

PAGE.
ARTICLE I.—Epilepsy, with a peculiar kind of Parasitic Animals in the Alimentary Canal. By G. W. Powell, M. D., of Childersburg, Alabama, ........ 127
ARTICLE II.—Medical Clinic for Session of 1860. By. J. G. Westmoreland, M. D., ........ 130
ARTICLE III.—Review of the Memorandica Medica; or, Note-book of Medical Principles. By V. H. Taliaferro, M. D., Columbus, Georgia, ........ 133
ARTICLE IV.—Items from a case book. By H. D. Capers, Atlanta, Georgia, ........ 140

### SELECTIONS.

Veratrum Viride, ........ 154
Tropical Dysentery, ........ 150
Rules for making good Wine, ........ 158
Anæsthesia and Anæsthetics, ........ 161

### EDITORIAL AND MISCELLANEOUS.

National Medical Association, ........ 170
Liquor Soda Tartras, ........ 172
Gleet, ........ 172
Errata, ........ 173
Notice of Leidy's Anatomy, ........ 173
Bibliographical, ........ 175
Druggists, ........ 179
Passage of a large Stone through the Urethra, ........ 182
Vaccination, ........ 183
Curious Case of Monomania, ........ 186
Medical Statistics of the U. S. Army, for 21 years, ........ 187
List of Payments, ........ 190
Meteorological Table, ........ 190

